# Genomic analysis of an extremophile PLS47 from a deep-sea vent

**DOI:** 10.3389/fmicb.2026.1767031

**Published:** 2026-02-25

**Authors:** Baiq Repika Nurul Furqan, Made Puspasari Widhiastuty, Febriani, Deviyanthi Nur Afifah, Teuku Mohamad Iqbalsyah, Akhmaloka

**Affiliations:** 1 Biochemistry and Biomolecule Engineering Research Group, Faculty of Mathematics and Natural Sciences, Institut Teknologi Bandung, Bandung, Indonesia; 2 Biomolecules Application Research Group, Chemistry Department, Faculty of Mathematics and Natural Sciences, Syiah Kuala University, Banda Aceh, Indonesia

**Keywords:** deep-sea vent, extremophile, genome, *Geobacillus thermocatenulatus*, PLS47

## Abstract

Indonesia's submarine hydrothermal vents represent underexplored ecosystems harboring thermophilic microbial diversity with significant biotechnological potential. In the study, the whole-genome sequencing, assembly, and comparative genomic analysis of strain PLS47, an extremophilic bacterium isolated from the Pria Laot hydrothermal vent, Sabang, Aceh, Indonesia is reported. The genome was sequenced using Oxford Nanopore Technology, assembled de novo, and polished to obtain a high-quality draft genome. Genome annotation identified genes associated with thermotolerance, stress response, and diverse metabolic pathways, including COG, KEGG, and CAZymes analysis. Comparative genomic analyses, including ANI and phylogenomic, confirmed its taxonomic placement and highlighted genomic distinctions from closely related terrestrial strains. Pangenome analysis further revealed unique gene sets potentially linked to adaptation to deep-sea hydrothermal environments. The complete genome sequence local isolate PLS47 is 3,772,236 bp at chromosomal genome with GC content at 52.02%, along with 56,806 bp at plasmid DNA with GC content at around 41.06%. A total of 3,708 coding sequences (CDSs) were identified, 6 rRNA (5S, 16S, and 23S), 27 rRNA, 88 tRNA genes, and 17 pseudogenes. A comparison of the genome to data based on Average Nucleotide Identity shows that the genome is closely related to the *Geobacillus thermocatenulatus*. Functional analysis revealed numerous enzyme-coding genes, including proteases, peroxidases hydrolases, esterases, dehydrogenase, hydratases, and lipases. In addition, the genome exhibits a number of stress-tolerant genes. Detailed analysis of the hydrolase genes, especially for lipolytic enzymes such as esterase and lipase, showed that the genome exhibits true lipase like putative lipase, monoacyl glycerol lipase (MAGL) motif and other lipase like GDSL-type esterase/lipase motif. The genomic information provides an understanding of thermophilic genomes and their relevance to stress-tolerant adaptation and explores potential genes, especially for industrial applications.

## 1 Introduction

Indonesia, situated along the Pacific Ring of Fire, hosts diverse geothermal environments that provide ideal habitats for extremophilic microorganisms. Numerous extremophiles have been isolated from sites including the Cimanggu hot spring, Papandayan Crater, Garut, Domas Crater, Tangkuban Perahu Mountain, Bandung, West Java (Widhiastuty et al., 2009), Hujan Crater hot, Manuk hot spring (Widhiastuty et al., 2011), and Wayang hot spring (Febriani et al., 2013). The majority of bacteria identified from hydrothermal vents have been recovered using culture-dependent approaches. Thermo-halophilic bacteria have been isolated from under sea fumarole in the Pria Laot Sabang area of Weh Island, Aceh, Indonesia. Several of these isolates produce hydrolytic enzymes, including α-amylase from isolate PLS75 (Iqbalsyah et al., 2018), lipase from isolate PLS80 (Febriani et al., 2019), and protease from isolate PLSA (Iqbalsyah et al., 2019). DNA polymerase I has also been cloned from isolate PLS80 (Febriani et al., 2018) and isolate PLSA (Iqbalsyah et al., 2020). Suzanni et al. (2018) also reported a bacterial isolate, PLS76, capable of producing antibiotics. Beyond the terrestrial systems, submarine hydrothermal vents represent promising but underexplored reservoirs of thermophilic microbial diversity.

The Pria Laot hydrothermal vent at Sabang remains one of the largely unexplored vent sites in the Indo-Pacific region. Its geochemical characteristics and thermal gradients provide a suitable environment for the discovery of novel thermophilic bacterial lineages. Several isolates, including PLS75, PLS76, PLS80, PLSA, and PLS47, have been obtained from this site (Iqbalsyah et al., 2020). Among them, strain PLS47 was successfully obtained from the Pria Laot hydrothermal vent and was found to be phylogenetically closely related to *Geobacillus thermocatenulatus* based on 16S rRNA gene analysis (Febriani, personal communication, 2023). Members of the genus *Geobacillus* are well known for their thermotolerance and their ability to produce thermostable enzymes with significant biotechnological potential (Hussein et al., 2015).

However, despite increasing interest in thermophilic *Geobacillus* species, genomic information from strains isolated from deep-sea hydrothermal vents remains extremely limited. To date, no complete genome sequence has been reported for strain PLS47 or for other thermophilic bacteria isolated from the Pria Laot vent system. Most available *Geobacillus* genomes originate from terrestrial or compost environments, such as *Pseudoxanthomonas taiwanensis* AL17 (Afifah et al., 2024), leaving the genetic basis of adaptation to deep-sea hydrothermal conditions largely unexplored.

In this study, we report the whole-genome sequence, assembly, and annotation of the extremophilic isolate PLS47. The genomic analysis revealed candidate genes potentially involved in thermotolerance, stress adaptation, and industrially relevant metabolic pathways. This study expands the current genomic landscape of extremophiles from hydrothermal vent ecosystems and highlights the potential of PLS47 as a valuable genetic resource from an underexplored Indonesian deep-sea environment in Indonesia.

## 2 Materials and methods

### 2.1 Growth of isolate PLS47

Bacterial culture was provided from a collection of thermophilic microorganisms isolated from deep sea vent in Pria Laot Sabang, Aceh (Iqbalsyah et al., 2020). The glycerol stock was incubated in liquid media at 70°C overnight. Subsequently, the culture was transferred to fresh ½ Thermus medium containing yeast extract (0.4% w/v; Oxoid, Hampshire, UK), peptone (0.8% w/v; Oxoid, Hampshire, UK), NaCl (2% w/v; Merck, Darmstadt, Germany), and glucose (0.25% w/v; Merck, Darmstadt, Germany) dissolved in seawater, then incubated at 70°C for 16–18 hours.

### 2.2 DNA extraction

DNA was extracted from liquid culture samples using the Quick-DNA-HMW MagBead Kit (Zymo Research, Irvine, CA, USA; Cat. No. D6060). In the DNA purification procedure, 400 μL of bacterial culture in a microtube was added to 400 μL of Quick-DNA™ MagBinding Buffer (Zymo Research, Irvine, CA, USA) and vortexed. Afterward, 33 μL of MagBinding Beads (Zymo Research, Irvine, CA, USA) were quickly added and resuspended five times. The sample was placed on a shaker at 1353 × g for 10 minutes. The sample was transferred to a magnetic stand and allowed to separate. The supernatant was discarded, while 500 μL of Quick-DNA™ MagBinding Buffer (Zymo Research, Irvine, CA, USA) was added to the pellet. The sample was resuspended five times and then shaken for 5 minutes at room temperature.

The sample was placed on a magnetic stand and allowed to separate. The supernatant was discarded, and 500 μL of DNA Pre-Wash Buffer (Zymo Research, Irvine, CA, USA) was added to the pellet and resuspended ten times. The sample was placed back on the magnetic stand until separation occurred. The supernatant was discarded, and 900 μL of g-DNA Wash Buffer (Zymo Research, Irvine, CA, USA) was added and resuspended 10 times. The sample was transferred to a new microtube, placed on the magnetic stand, and the supernatant discarded. The washing step with 900 μL g-DNA Wash Buffer (Zymo Research, Irvine, CA, USA) was repeated. The pellet was dried at room temperature for 20 minutes. After that, 50 μL of DNA Elution Buffer (Zymo Research, Irvine, CA, USA) was added and resuspended 20 times, then incubated at room temperature for 5 minutes. The sample was placed on the magnetic stand until separation, and the DNA was transferred to a new microtube. DNA was stored at ≤ −20°C. The quantity and quality of the extracted DNA were measured using a NanoDrop spectrophotometer (Thermo Scientific, Wilmington, DE, USA; Cat. No. ND-8000-GL) at 260 nm, a Qubit fluorometer (Thermo Fisher Scientific, Waltham, MA, USA), and agarose gel electrophoresis (agarose; Invitrogen, Carlsbad, CA, USA).

### 2.3 Genome sequencing

The genome was sequenced using a kit from Oxford Nanopore Technologies (ONT), following the manufacturer's protocol. Based on the protocol, at the end preparation stage, volume of 45 μL to 1 μg of DNA (genomic DNA, amplicon or cDNA) in a microtube is added with a solution that has been prepared in the kit, namely Ultra II End-prep reaction buffer as much as 7 μL, Ultra II End-prep enzyme mix as much as 3 μL and Nuclease-free water (NFW) as much as 5 μL. The mixture is vortexed and transferred to a 0.2 mL PCR tube, then incubated for 5 min at 20 °C and for 5 min at 65 °C. The mixture was transferred into a 1.5 mL Eppendorf DNA LoBind tube provided in the kit, and 60 μL of AMPure XP beads were added. Afterward, it was incubated on a shaker for 5 min. The mixture was placed on a magnetic stand until the supernatant and pellet separated. The supernatant was discarded, while 200 μL of wash beads (70% ethanol in NFW) was added to the pellet. The wash beads were discarded thoroughly, and the sample was dried at room temperature. Afterward, 31 μL of NFW was added to the sample and incubated for 2 min at room temperature. The sample was placed back on the magnetic stand until the supernatant and pellet separated. The NFW was discarded, and 31 μL of the pellet was added to a new Eppendorf DNA LoBind tube. Approximately 30 μL of the sample was ready to be inserted into the ligation adapter. In the adapter ligation stage, 30 μL of the sample was added to 20 μL of the Adapter Mix and 50 μL of the Blunt/TA Ligation Master Mix and vortexed. Afterward, the sample was incubated for 10 min at room temperature. The sample was prepared for sequencing on the GridION sequencer using MinKNOW v21.11.17 software.

### 2.4 Assembly quality metrics

The first assembly quality assessment involved base calling, which translates raw signals generated by the sequencer into nucleotide sequences. Base calling analysis was performed using ONT Guppy v5.1.13 in high accuracy mode (Wick et al., 2019). Reads were then corrected and trimmed using Nanoplot v1.40.0 (De Coster et al., 2018). The consensus sequence was checked through *De novo* Assembly using Flye v2.8.3 (Kolmogorov et al., 2019). Genome sequence polishing was carried out several times, with four rounds of Racon v1.5.0. and one round of Medaka v.1.5.0 (Vaser et al., 2017). Sequence mapping was performed using minimap2 (Parks et al., 2015), and sequence quality was determined using Quast v55.0.2 (Gurevich et al., 2013). Then, annotation was performed using the NCBI PGAP pipeline to assess the similarity of the sequences obtained from the NCBI (Tatusova et al., 2016). Closely related or nearly similar species to the sequences obtained were then identified using CheckM from dFast-QC v0.2.1 (Li, 2018). Genome visualization was performed using Circos (Gurevich et al., 2013).

### 2.5 Gene annotation

Genome annotation (PLS47, *G. thermocatenulatus* KCTC, and *G. thermocatenulatus* BGSC 39A1) was performed using Prokka v1.14.5 (Seemann, 2014). The Kyoto Encyclopedia of Genes and Genomes (KEGG) annotation was conducted using BlastKOALA (Kanehisa et al., 2016). Clusters of Orthologous Groups (COG) profiles were generated using DIAMOND BLASTp v0.9.24 (Buchfink et al., 2015) against the COG database (Galperin et al., 2020). AntiSMASH v7.1.0 was used to identify and annotate biosynthetic gene clusters in the genomes (Blin et al., 2023). The carbohydrate-active enzyme (CAZy) prediction was performed using the dbCAN3 meta-server (Zheng et al., 2023).

### 2.6 Genome visualization

Circular visualizations of the genome and plasmids were created using Circos v0.69.8 (Krzywinski et al., 2009).

### 2.7 Pairwise genome sequence comparison

For phylogenomic analysis, all pairwise genome comparisons were carried out using the Genome BLAST Distance Phylogeny (GBDP) approach, with intergenomic distances inferred using the “trimming” algorithm and distance formula d5 (Meier-Kolthoff et al., 2013). A total of 100 distance replicates were calculated. Digital DNA–DNA hybridization (dDDH) values and confidence intervals were estimated using the default settings of GGDC 4.0 (Meier-Kolthoff et al., 2022). Additionally, genomic similarities between strain PLS47 and closely related species were assessed using the average nucleotide identity (ANI) algorithm implemented in FastANI v1.32 (Jain et al., 2018), and the results were visualized using the ggplot2 package in R (Wickham, 2016).

### 2.8 Phylogenetic inference

The inferred intergenomic distances were used to construct a balanced minimum evolution tree with branch support calculated using FASTME v2.1.6.1, including SPR postprocessing (Lefort et al., 2015). Branch support was estimated from 100 pseudobootstrap replicates. The trees were midpoint rooted (Farris, 1972) and visualized using the ggtree package (Yu et al., 2017).

### 2.9 Pangenome analysis

Prior to pangenome analysis, all reference genomes (Table 1) were re-annotated with Prokka v1.14.5 (Seemann, 2014) after being downloaded from RefSeq NCBI. Pangenome analysis was performed using Roary v3.13.0 (Page et al., 2015), with a minimum BLASTp identity threshold of 90%. Single representative sequences from each pangenome cluster were aligned against the KEGG database using GhostKOALA (Kanehisa et al., 2016) and the COG database (Galperin et al., 2020) using DIAMOND BLASTp v0.9.24 (Buchfink et al., 2015).

**Table 1:** List of genomes used for comparative analysis.

**Species**	**Accession numbers**	**Genome size**	**G+C content**	**Assembly level**	**Number of genes**	**Habitat/environmental**	**Utilized for analysis**
*G. thermocatenulatus* KCTC 3921	PRJNA353561	3,742,258 bp (chromosome only)	52%	Complete/Circular	3,772	Hot-gas well	Phylogenomic, ANI, and PangenomAnalysis
*G. thermocatenulatus* BGSC 93A1	PRJNA212540	1,722,151 bp and 1,841,649 bp	52%	Contig	3,685	Oilfield	Phylogenomic, ANI, and PangenomAnalysis
*G. zalihae* NBRC 101842	PRJDB415	3.5 Mb	52%	Contig	3,698	–	Phylogenomic and ANI Analysis
*G. thermoleovorans* KCTC 3570	PRJNA310809	Chromosome = 3,450,609 bp Plasmid = 48,708 bp	52.5%	Complete/Circular	Chromosome = 3,521	Soil near hot water effluent	Phylogenomic, ANI, and PangenomAnalysis
*G. thermopakistaniensis* MAS1	PRJNA222590	3.5 Mb	52%	Contig	3,767	Hot springs	Phylogenomic and ANI Analysis
*G. kaustophilus* NBRC 102445	PRJNA531185	3,670,957 bp	52%	complete/circular	3,815	–	Phylogenomic and ANI Analysis
*Geobacillus* sp. G4	PRJNA1124973	3.4 Mb	52.5%	Contig	3,460	Sediment	Phylogenomic and ANI Analysis
*G. proteiniphilus* 1017	PRJNA353982	3.6 Mb	52%	Contig	3,813	Production water	Phylogenomic and ANI Analysis
*G. jurassicus* NBRC 107829	PRJDB428	3.5 Mb	52%	Contig	3,606	–	Phylogenomic and ANI Analysis
*G. stearothermophilus* ATCC 12980	PRJNA212538	2.6 Mb	53%	Contig	2,815	Spoiled canned food	Phylogenomic and ANI Analysis
*G. subterraneus* KCTC 3922	PRJNA310054	3,474,426 bp	52%	Complete/Circular	3,384	Liaohe oil field	Phylogenomic and ANI Analysis
*G. icigianus* G1w1	PRJNA246135	3.6 Mb	51.5%	Contig	3,704	Hot spring sediments	Phylogenomic and ANI Analysis
*Geobacillus subterraneus aromaticivorans* DSM 23066	PRJNA632297	3.6 Mb	52%	Contig	3,708	–	Phylogenomic and ANI Analysis
*Geobacillus* sp. CX412	PRJNA871097 CP103461-CP103464.	Chromosome = 3,440,897 bp Plasmid 1 = 78,249 bp Plasmid 2 = 20,896 bp Plasmid 3 = 20,783 bp	42.5 %	Complete/Circular	3,763	Estuarine mud	Phylogenomic, ANI, and PangenomAnalysis
*G. stearothermophilus* H6	PRJNA224116	3,054,993 bp	51.66%	Complete/Circular	3,750	Hyperthermophilic compost	Phylogenomic, ANI, and PangenomAnalysis
*G. stearothermophilus* GF16	PRJNA947575	3,319,893 bp	51%	Complete/Circular	3,527	Soil hydrothermal volcanic	Phylogenomic, ANI, and PangenomAnalysis
*G. kaustophilus* HTA426	PRJNA13233	3,544,776 bp	52%	Complete/Circular	3,656	Deepest Ocean	Phylogenomic, ANI, and PangenomAnalysis
*G. zalihae* JS1-2	PRJNA989262	3,532,984 bp chromosome 34,853 bp plasmid	52%	Complete/Circular	3,587	Compost	Phylogenomic, ANI, and PangenomAnalysis

## 3 Results and discussion

### 3.1 Complete genome sequence of PLS47

**Figure 1:**
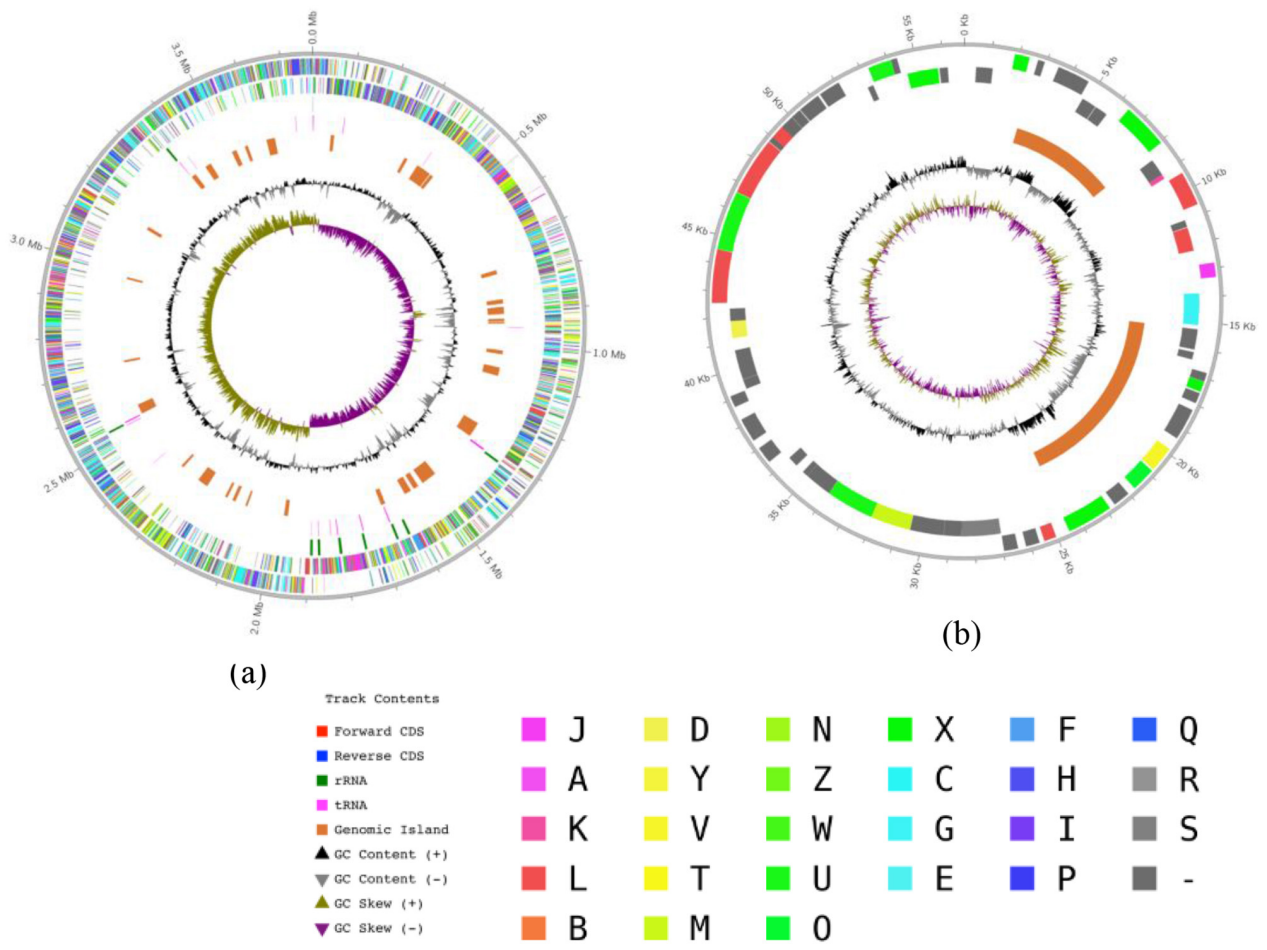
Genome map of the PLS47: **(a)** chromosome and **(b)** plasmid. From outer to inner: scale marks in kb. GC%, coverage, gene category, and COG category, respectively.

PLS47 is a Gram-positive rod-shaped bacterium with an optimum growth at 70 °C. The chromosomal genome of PLS47 is 3,772,236 bp in length, with an average G+C content is 52.02%, and contains 88 tRNA genes and 27 rRNA genes (Table 2 and Figure 1a). A total of 3,645 protein-coding regions were predicted in the chromosome of the genome (Table 2). The G+C content value and the amount of tRNA and rRNA in PLS47 are within the range of values found in *Geobacillus*, where the chromosomal G+C content ranges from 50% to 57% (Tanaka et al., 2025). Apart from the chromosomes, the PLS47 genome is distinct from other reported *Geobacillus* genomes in that it contains a plasmid. Its plasmid is 56,806 bp in length, with an average G+C content of 41.06% and 63 predicted protein-coding regions (Table 2 and Figure 1b). In the genome, most *Geobacillus* strains isolated from different environments do not have plasmids; however, *Geobacillus* strains isolated from deep-sea vents are found to have 1 or even 3 plasmids, whereas others do not contain any plasmids (Wissuwa et al., 2016).

**Table 2:** Genome features of the strain PLS47.

**Genome features**	**Chromosome**	**Plasmid**
Genome size (bp)	3,772,236	56,806
G+C content (bp)	52.02	41.06
CDSs	3.645	63
rRNA	27	0
tmRNA	1	0
tRNA	88	0

Analysis of the complete genome of PLS47 revealed the presence of plasmids, a feature that has been reported previously but remains rare among published complete genomes of *Geobacillus* and related species. The detection of this extrachromosomal element is noteworthy, as plasmid-free genomes appear to be typical of most available *Geobacillus* isolates. This contrast emphasizes the highly dynamic nature of plasmid evolution within bacterial lineages and suggests that PLS47 may have experienced different ecological or evolutionary pressures compared with strains residing in hot gas wells, oil fields, compost, or other environments (Sung et al., 2024).

Plasmids are widely understood as extrachromosomal elements carrying accessory genes, distinct from chromosomal core genes, and often provide adaptive advantages in specific or fluctuating environments (West-Eberhard, 1989). PLS47 inhabits an extreme ecosystem, namely a deep-sea vent, which may have driven its evolution through the acquisition of plasmids. The plasmids within its genome may enhance survival by providing metabolic pathways, stress tolerance, or mechanisms to cope with geochemical variability (Ciuchcinski et al., 2024). The absence of plasmids in other *Geobacillus* may reflect the historical loss of plasmids in environments where light stress did not favor their maintenance. Plasmid persistence is influenced by the interaction between environmental selection, host genomic background, and horizontal gene transfer (Brockhurst and Harrison, 2022). This may explain why PLS47 harbors a plasmid, whereas closely related phylogenetic relatives do not.

### 3.2 Phylogenomic relationships and genomic similarity

**Figure 2:**
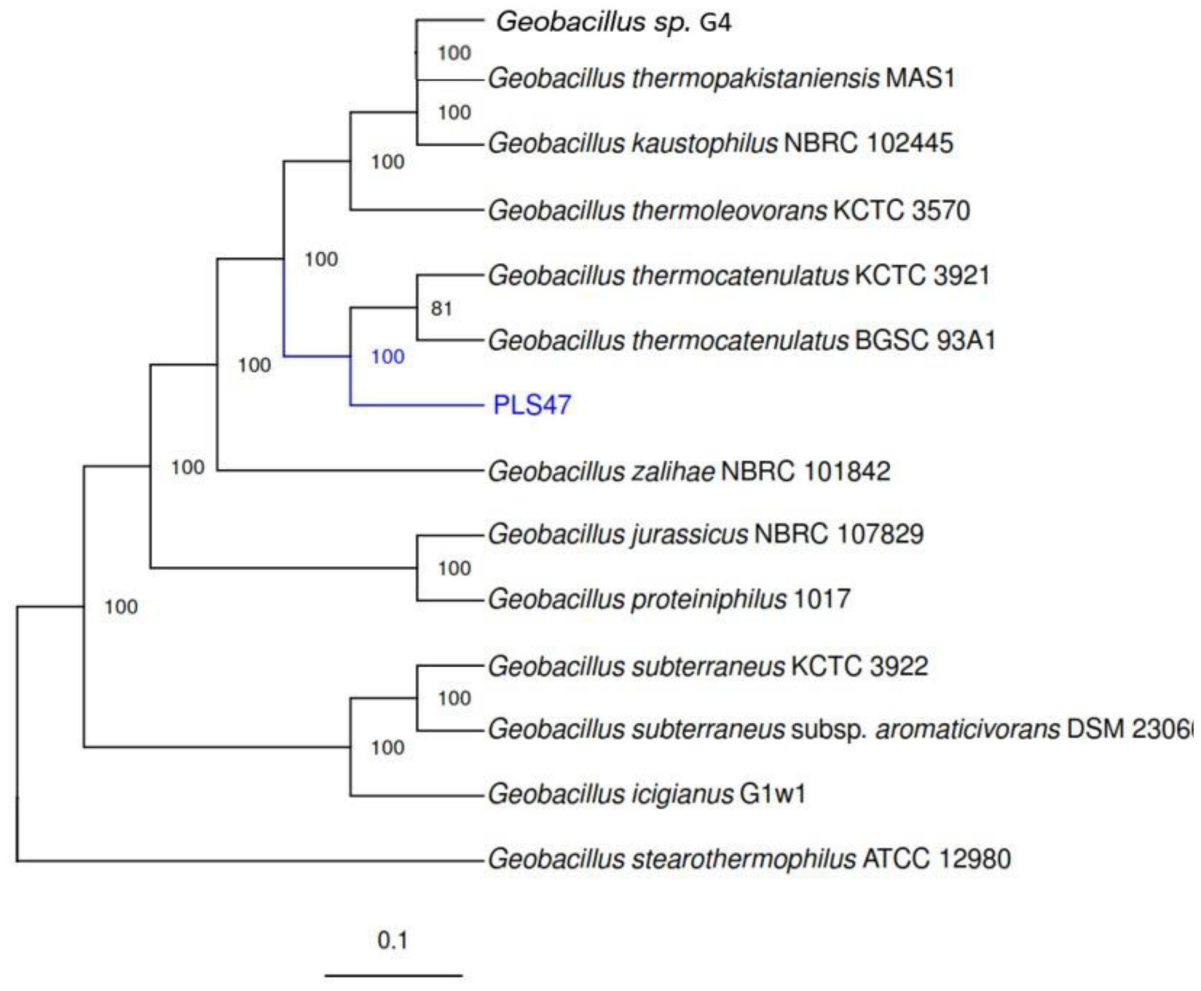
Phylogenomic analysis of strain PLS47 based on genome-wide comparison with other *Geobacillus* genomes.

Phylogenomic analysis of the genome was carried out using the genomic sequences from other *Geobacillus* species (Figure 2). Out of 120 available species, only 13 (Table 1) were selected because they possess a complete genome sequence. Furthermore, according to the NCBI Gene Bank (National Center for Biotechnology Information), only three species of *G. thermocatenulatus* have complete assembly-level genomes. The genome assembly process is complex and susceptible to sequence gaps and assembly-related genetic variation. The results showed that the PLS47 is closely related to the genome of *G. thermocatenulatus* KCTC 3921 and the *G. thermocatenulatus* BGSC 39A1 (Figure 2).

**Figure 3:**
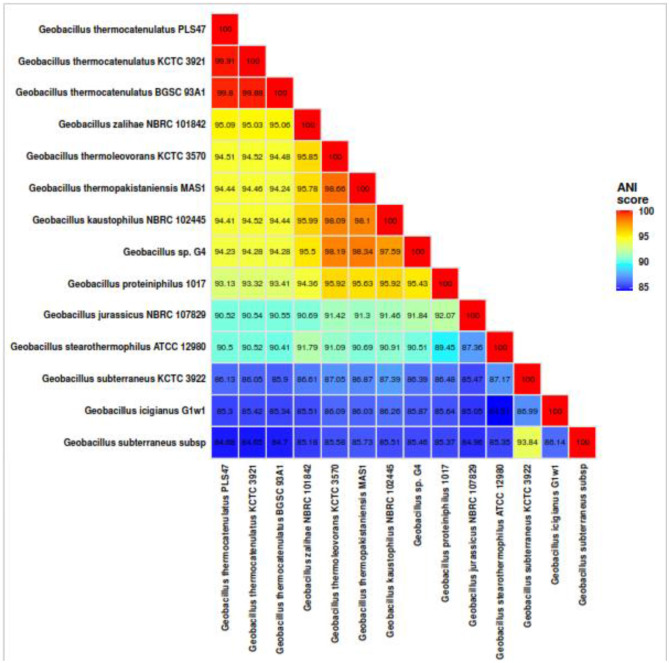
Pairwise comparisons of the PLS47 against closely related species based on average nucleotide identity (ANI).

ANI analysis performed using Dfast-qc 20 was used to compare the PLS47 genome with other genomes. The result showed that PLS47 is closely related to the genome of *G. thermocatenulatus* KCTC 3921 and the *G. thermocatenulatus* BGSC 39A1 (Figure 3). These results are in agreement with previous data based on the 16S rRNA gene. ANI analysis clearly supports the assignment of PLS47 to *G. thermocatenulatus*, as evidenced by ANI values of 99.9% and 99.8% with reference strains KCTC 3921 and BGSC 93A1, respectively. These values are well above the widely accepted species threshold of 95%−96%. In contrast, ANI values between PLS47 and other *Geobacillus* species were below the cutoff, including a borderline value of 95.01% with *G. zalihae*. This pattern highlights clear genomic separation between *G. thermocatenulatus* and other members of the genus, while also reflecting the close evolutionary relationships and genomic continuity commonly observed among thermophilic *Geobacillus* species. The results confirm the species-level identity of PLS47 and underscore the usefulness of ANI for resolving taxonomic relationships within the genus.

### 3.3 Extremophilicity of PLS47

**Figure 4:**
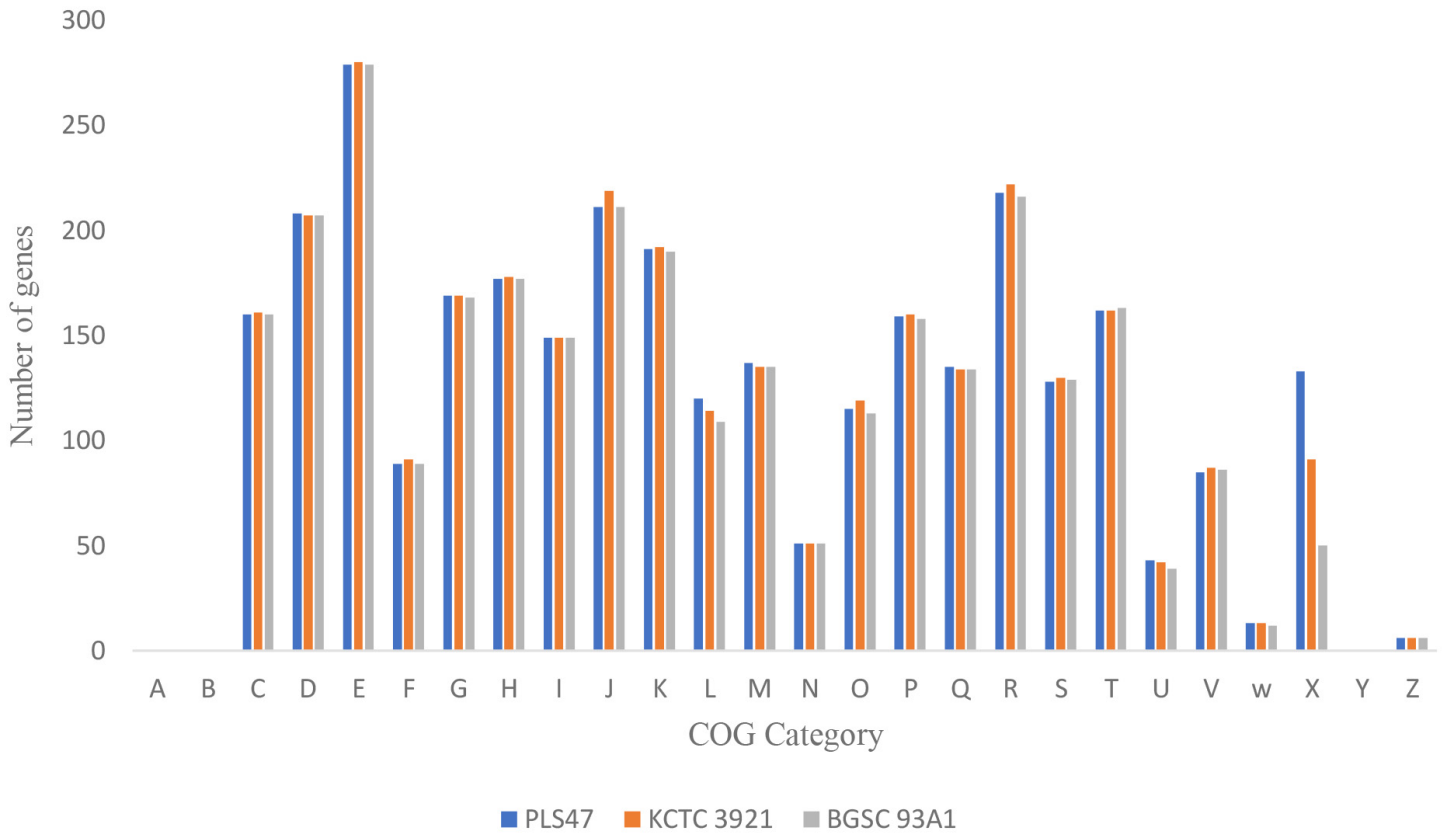
Number of genes associated with general COG functional categories. PLS47, *G. thermocatenulatus* KCTC 3921, and *G. thermocatenulatus* BGSC 39A1.

COG analysis identified 4,211 genes in the PLS47 genome (Figure 4). Of these, 27.86% (1,173 out of 4,211) were not found in the COG category, indicating the presence of novel genes in this organism. Furthermore, 3.04% of the genes (128 out of 4,211) were categorized as S genes (signifying genes with unknown functions). Meanwhile, 5.01% of the genes are categorized as J genes, related to play a key role in translation, ribosome structure, and biogenesis. Several bacteria isolated from deep-sea vents exhibit a high abundance of COG categories related to amino acid metabolism (E; 6.63%), energy production/conversion (C; 3.8%), transport of inorganic ions (P; 3.78%), and carbohydrate transport and metabolism (G; 4.01%). In contrast, the PLS47 genome did not show A (RNA processing and modification) and B (chromatin structure and dynamics) category genes, but showed Z category gene (cytoskeleton; 0.14%) and W category gene (extracellular structures; 0.31%).

COG analysis of other *Geobacillus* genomes revealed the absence of genes in categories A (RNA processing and modification) and B (chromatin structure and dynamics) (Figure 4). These results are consistent with typical prokaryotic genomes that lack complex chromatin and RNA transport systems like eukaryotes (Tatusova et al., 2016). Similarly, COG analysis of the *P. taiwanensis* AL17 genome showed no category A genes and only a single gene in category B (Afifah et al., 2024). This absence may reflect the streamlined nature of the PLS47 genome, optimized for survival in extreme environments. While PLS47 and *Geobacillus* sp. 12AMOR1 differ in certain gene counts, both share core COG functional categories related to energy production (C), defense mechanisms (V), amino acid transport (E), and secondary metabolite biosynthesis (Q) (Wissuwa et al., 2016). This conservation highlights the organism's adaptability to high temperatures, pressure, salinity, and heavy metal exposure (Galperin et al., 2015).

To further probe the characteristics of PLS47, the amount and metabolic potential of PLS47 and other *Geobacillus* with the whole genome sequence similarity were analyzed based on levels of COG and CAZymes. The results showed that there are several genes that encoded protein associated with stress tolerance, such as superoxide dismutase (SOD), which plays an important role in enabling cells to withstand high-temperature stress, and Cu/Zn superoxide dismutase (SOD1) enzyme, which functions under Cu^2+^/Zn^2+^ conditions (Table 4). At the same time, the ClpP protein was found to affect the temperature resistance of the strain (Gerth et al., 2008). The presence of the gene might make cells resistant to high temperatures (Askwith et al., 1994). As a thermophilic isolate, PLS47 should have genes related to high-temperature tolerance. The results showed that PLS47 had related genes for SOD, SOD1, and ClpP proteins; the related genes generally existed in the near-derived strains. The existence of related genes suggests the reasons for the high-temperature resistance of PLS47.

The genome of PLS47 also encodes the chaperonin groL and groS (Table 3), which are essential for proper protein folding under stress conditions. groL forms a tetradecameric barrel that provides a protected environment for unfolded or misfolded proteins, while groS acts as a co-chaperonin “cap,” facilitating ATP-dependent folding cycles (Horwich et al., 2006). The presence of these chaperonins is particularly relevant given the extreme environment of PLS47, which includes high temperature, pressure, salinity, and exposure to heavy metals. By ensuring correct protein folding and preventing aggregation, groL and groS likely enhance cellular stress tolerance and maintain the functionality of metabolic and structural proteins, enabling PLS47 to survive and thrive under such harsh conditions. Similar roles of groL/groS in stress adaptation have been documented in thermophilic and barophilic bacteria, highlighting their importance in extremophilic environments (Hayer-Hartl et al., 2016). As a thermophilic bacterium, the PLS47 strain should contain genes related to high-temperature tolerance. The presence of related genes suggests the reason for the high-temperature resistance of PLS47. In contrast to other *Geobacillus* genomes and the *P. taiwanensis* genome, groS and groL were not found in the genome, but one of them was found with the name groEL or groES gene (Afifah et al., 2024).

**Table 3:** Stress tolerance related to genes of PLS47.

**ID features**	**Genes**
Heat adaptation and response	SOD superoxide dismutase (SOD1) enzyme Cu/Zn under Cu^2+^/Zn^2+^ ClpP protein Spermidine/putrescine-binding periplasmic genes DNA gyrase subunit A (EC 5.99.1.3) Heat adaptation Heat responses Oxidative stress DNA gyrase subunit B (EC 5.99.1.3) S-adenosylmethionine decarboxylase proenzyme (EC 4.1.1.50)
Stress extreme environment adaptation	groL groS Transposase InsG Transposase family ISAfl1 DNA-binding protein HU Spermidine synthase (EC 2.5.1.16) Arginine decarboxylase (EC 4.1.1.19). tmRNA-binding protein SmpB Translation elongation factor LepA Heat-inducible transcription repressor HrcA Heat shock protein GrpE Chaperone protein DnaK Chaperone protein DnaJ
Oxidative and osmotic stress-tolerant	Ribosomal protein L11 methyltransferase (EC 2.1.1.-) Ribosome-associated heat shock protein implicated in the recycling of the 50S subunit (S4 paralog) Ribonuclease PH (EC 2.7.7.56) ATP-dependent HSL protease ATP-binding subunit HslU Catalase (EC 1.11.1.6) Peroxidase (EC 1.11.1.7) NAD-dependent protein deacetylase of the SIR2 family Thioredoxin reductase (EC 1.8.1.9) Osmotic stress (proVWXSBA, fadANM, betBA, trkAH, opuBDCA, opcR, putP, yrgG, kch, and nhaC) Ectoine biosynthesis (ectCBAD) oxidative stress (hmp, pfpI, Usp, katE, and osmC) Cold-shock protein (cspA) Heavy metals such as arsenic (arsRBC), cobalt (czcD, ecfT, ecfA1, ecfA2), zinc (zupT, yqgT, rseP, czrA, znuACB, zurR, nprE, qor, sprL), cadmium (zntA), magnesium (corA), molybdenum (modABC), copper (copZA, csoR, copB, cutC, ycnK), and manganese (mntCBA) Antibiotics (norM, bacA, lmrB, fsr, pbp1b, ykkDC, and yitG), and fluoride resistance (crcB).

The ability of *Geobacillus* to thrive in diverse and often harsh environments may be due to the predicted coding transposons of many *Geobacillus* species (Bouzas et al., 2006; Bergquist et al., 2014). The number of predicted coding transposons reflects the chromosomal variability of an organism; these might add or delete non-essential genes or gene clusters based on environmental conditions, representing the organism's ability to adapt to the environment (Brumm et al., 2016). As shown in Table 4, the PLS47 contains 212 predicted coding transposons. After comparing with closely related strains and reviewing the literature (Brumm et al., 2016), it was found that the number of predicted transposons encoded by the PLS47 is three times higher when compared with the *G. thermocatenulatus* KCTC 3921 and the *G. thermocatenulatus* BGSC 93A1. At the same time, the number of predicted transposons encoded by PLS47 in the *Geobacillus* genus is not significantly different from that of *Geobacillus* sp. 12AMOR1 (Wissuwa et al., 2016), which is one of the *Geobacillus* that was also isolated from deep-sea vent sediment. This suggests that PLS47 shows a strong ability to adapt to environmental change.

**Table 4:** Comparison of transposons.

**Transposase**	**PLS47**	***G. thermocatenulatus* KCTC 3921**	***G. thermocatenulatus* BGSC 93A1**
Total	212	74	85

In addition, the genome also contains the spermidine/putrescine-binding periplasmic genes (Table 3), probably responsible for the import of the spermidine/putrescine ABC transporters and related to the thermophilicity of the isolate. The gene plays a role in adaptation to environmental stress, including heat, through regulation of cell homeostasis and molecular stability (Zhang et al., 2025). A periplasmic component of the ABC transporter system that imports the polyamines spermidine and putrescine into bacterial cells. Polyamines (spermidine, putrescine) are positively charged molecules that bind to DNA, RNA, and proteins and play a key role in stabilizing macromolecular structures, protecting against oxidative stress, and regulating growth and replication (Qiao et al., 2024). The spermidine/putrescine ABC transporters are also found in the genome of *Geobacillus* sp. 12AMOR1 that is isolated from a deep-sea vent.

### 3.4 Comparison with other *Geobacillus* genomes

**Figure 5:**
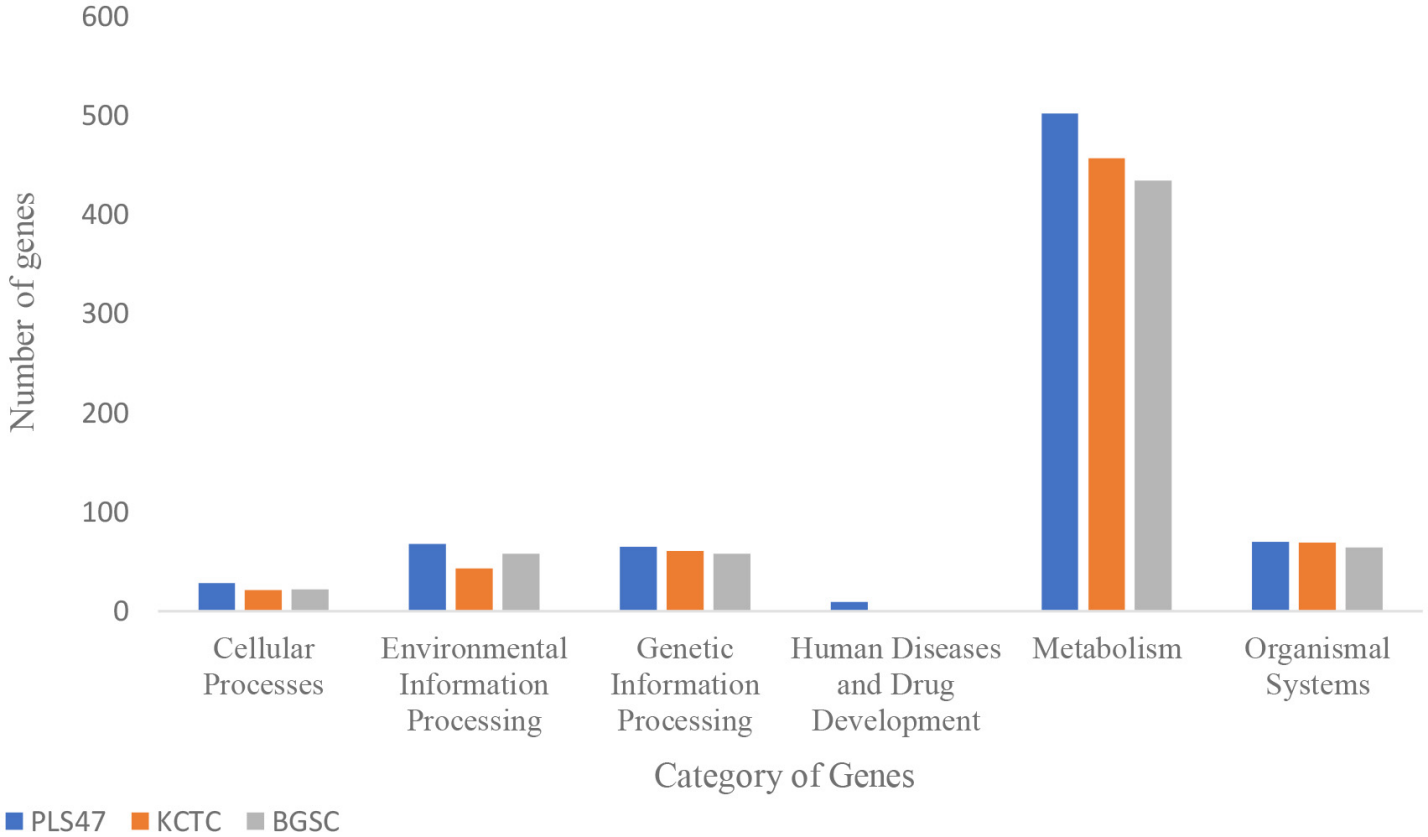
Histogram of KEGG pathway categories. PLS47, *G. thermocatenulatus* KCTC 3921, and *G. thermocatenulatus* BGSC 39A1.

The Kyoto Encyclopedia of Genes and Genomes (KEGG) is a comprehensive database of biological systems that integrates genomic, chemical, and functional information across biological systems. KEGG GENES collects all known complete genome sequences, including the minimum information for each gene. The KO system (KEGG ORTHOLOG) connects different KEGG annotation systems. Following KO annotation, KEGG metabolic pathway classification is performed based on the relationships between KO identifiers and KEGG pathways. There are seven categories: cellular processes, environmental information processing, genetic information processing, human diseases, metabolism, organism systems, and drug development. The complete genomes of the *Geobacillus* were discovered and compared. To analyze the metabolic pathway of PLS47, genes from PLS47 and two closely related strains were compared against the KEGG functional pathway database for functional annotation (Figure 5).

The proportion of six functional genes of PLS47 was 3.45% (128 genes; cellular processes), 12.3% (456 genes; environmental information processing), 9.8% (365 genes; genetic information processing), 1.5% (56 genes; human diseases), 70.9% (2,632 genes; metabolism), and 1.94% (72 genes; organismal systems). It was indicated that there are six categories of functional genes of PLS47 (excluding drug development). At the same time, Figure 5 showed that the metabolic functions of PLS47 are mainly associated with carbohydrate and amino acid metabolism. The metabolic function of the PLS47 involves a significantly higher number of genes compared with those of other closely related strains (>50%−100%). It was revealed that the PLS47 exhibits the most robust metabolism in the genus *Geobacillus*. In addition, the PLS47 contains genes assigned to the human disease category, whereas such genes were not detected in the other strains. The genes annotated to the antibiotic resistance pathway include norM, bacA, lmrB, fsr, pbp1b, ykkDC, and yitG, as well as the ectoine biosynthesis pathway (ectCBAD), which has genes associated with antibiotic resistance and more active metabolism (Figure 5). It also implies that the PLS47 has a broader range of applications. *Geobacillus* sp. 12AMOR1, isolated from a deep-sea vent, contains genes associated with human disease. Specifically, genes annotated to the β-lactam resistance pathway have been associated with human disease (Wissuwa et al., 2016).

**Figure 6:**
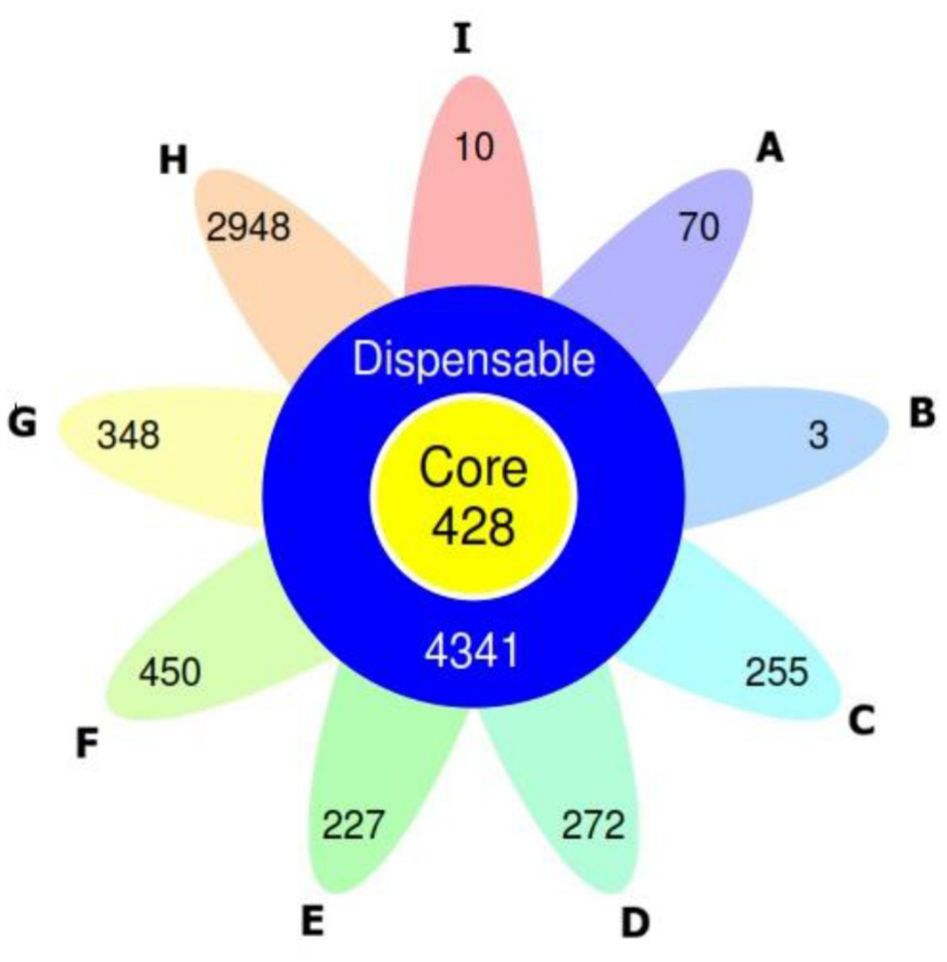
Flower plot representing the number of core and strain-specific genes of *Geobacillus* species based on clusters of orthologous groups. A = PLS47; B = *G. thermocatenulatus* KCTC 3921; C = *G. thermoleovorans* KCTC 3570; D = *G. zalihae* JS1-2; E = *G. kaustophilus* HTA426; F = *G. stearothermophilus* H6; G = *G. stearothermophilus* GF16; H = *Geobacillus* sp. CX412; I = *G. thermocatenulatus* BGSC 93A1.

In addition to the above data, the PLS47 was also compared with other *Geobacillus* using pan-genome analysis. Pan-genome is the combination of all genes found in multiple strains of a single species or genus. A pan-genome consists of core genes (found in all strains), accessory or dispensable genes (found in some but not all strains), and unique genes (found in only one strain). Genomic comparisons of PLS47 (isolated from a deep-sea hydrothermal vent) and strains of *G. thermocatenulatus* (KCTC 3921 from a hot gas well and BGSC 39A1 from an oilfield), alongside several other *Geobacillus* species, reveal compelling evidence of ecological and evolutionary differentiation driven by habitat extremes. Pan-genome analysis discerned that PLS47 possesses a notably elevated number of 70 unique genes, whereas KCTC 3921 and BGSC 39A1 carry only 3 and 10 unique genes, respectively (Figure 6).

The extreme deep-sea vent environment imposes intense selective pressures that require specialized physiological adaptations, which may account for the high number of unique genes identified in PLS47 (70 genes). The genes are likely involved in tolerance to high hydrostatic pressure, detoxification of heavy metals or sulfur compounds, and protein or membrane stabilization mechanisms under high temperature and hypersaline conditions. In contrast, strains KCTC 3921 and BGSC 39A1 originate from less extreme, chemically and pressure-sensitive habitats, which may account for their significantly lower number of unique genes (Wang et al., 2020).

The unique genes of each strain are summarized in Table 5. PLS47 has 32 genes with known functions and 38 hypothetical proteins with unknown functions. KCTC 3921 contains one gene with a known function and two hypothetical proteins, while BGSC 39A1 contains two genes with known functions and eight hypothetical proteins. For *G. thermocatenulatus* KCTC 3921, a unique gene encoding Ni/Fe hydrogenase was identified; this enzyme plays a key role in the oxidation of hydrogen to produce energy and is commonly found in high-pressure gas environments, such as hot gas wells (Mohr et al., 2018). In *the G. thermocatenulatus* BGSC 39A1 genome, a unique gene encoding *cytochrome o ubiquinol oxidase* was detected; this enzyme assists in aerobic respiration under high-aeration conditions (Chepuri et al., 1990). Although oilfield environments are generally anaerobic, the presence of this gene suggests potential respiratory flexibility. Such capacity may allow the *G. thermocatenulatus* BGSC 39A1 to tolerate transient oxygen exposure or exploit localized microaerobic niches, which can arise during oilfield operations, including fluid injection, mixing processes, or redox heterogeneity (Liebensteiner et al., 2014). Another unique gene encoding a *transcriptional activator* is related to environmental stress tolerance and may aid survival under harsh chemicals or temperature stresses in oilfield environments (Peltek et al., 2024).

**Table 5:** Comparison of unique genes in the PLS47 genome, *G. thermocatenulatus* KCTC 3921, and *G. thermocatenulatus* BGSC 39A1 genome.

**PLS47**	***G. thermocatenulatus* KCTC 3921**	***G. thermocatenulatus* BGSC 39A1**
IS701 family transposase ISAfl1	Ni-Fe Hydrogenase (hyaB)	Cytochrome o ubiquinol oxidase (cyoA)
Spermidine/putrescine-binding periplasmic	Hypothetical protein (2)	Transcriptional activator (toxR)
norM, bacA, lmrB, fsr, pbp1b, ykkDC, and yitG		Hypothetical protein (8)
Linearmycin resistance ATP-binding protein LnrL		
putative protein YhaP		
ectCBAD		
Putative efflux system component YknX		
Putative ABC transporter ATP-binding protein YknY		
Putative ABC transporter permease YknZ		
Replication-relaxation		
FtsK/SpoIIIE family protein		
Staphylococcal nuclease homolog		
Bacteriophage holin family protein		
Siphovirus ReqiPepy6 Gp37-like protein		
Phage tail protein RIFT-related domain protein		
3'5′ exonuclease DinG		
Transposase InsG		
Hydroxyacylglutathione hydrolase		
SPbeta prophage-derived uncharacterized protein YolA		
Type-2 restriction enzyme BamHI		
Helix-turn-helix		
Modification methylase BamHI		
Acetyltransferase (GNAT) domain protein		
Enterobactin exporter EntS		
Transposase		
Bacitracin transport ATP-binding protein BcrA		
ABC-2 family transporter protein		
MobA/MobL family protein		
Type IV secretory system conjugative DNA transfer		
DNA topoisomerase 3		
groL chaperonin		
groS chaperonin		
Hypothetical protein (32)		

PLS47 contains 32 unique genes with known functions, all of which encode enzymes that contribute to its resistance to extreme deep-sea vent environmental stress, specifically the *groL* and *groS* chaperonin genes, as well as transposase genes. Although PLS47 showed a very high level of phylogenomic and ANI data close to *G. thermocatenulatus* KCTC 3921, followed by *G. thermocatenulatus* BGSC 39A1, other data provide information on the differences between PLS47 and the other *G. thermocatenulatus*, thus indicating that PLS47 is a unique strain with potential for further exploration.

### 3.5 Genome and potential genes in PLS47

**Figure 7:**
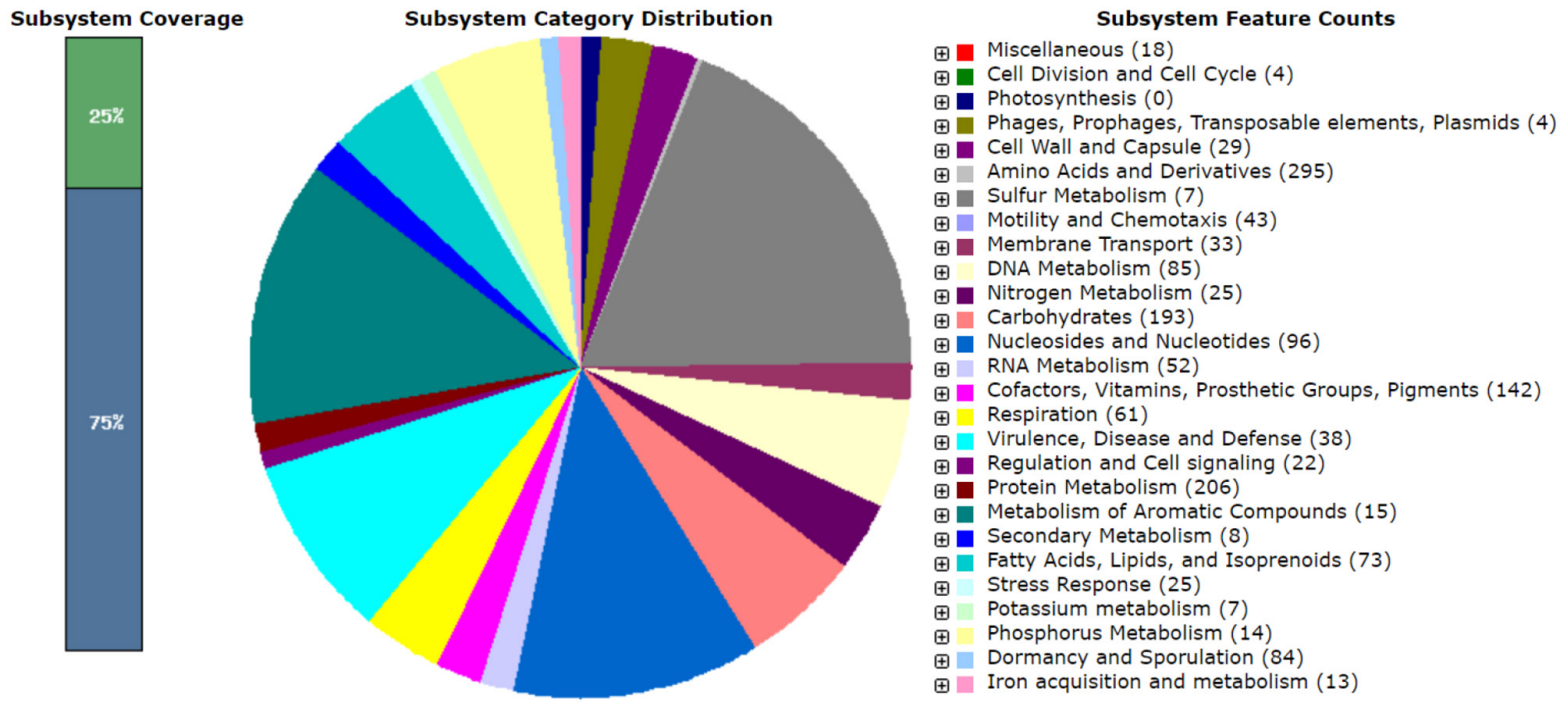
Overview of the subsystem categories of genome PLS47 using the RAST server.

Further analysis using the RAST program showed the presence of several genes encoding potential enzymes (Figure 7). Some of the enzymes include hydratases, dehydrogenases, peptidases, hydrolases, lipases, proteases, peroxidases, and esterases (Table 6). Detailed analysis of the hydrolase genes, especially for lipolytic enzymes, such as esterases and lipases, showed that the genome exhibits true lipase-like monoacyl glycerol lipase (MAGL motif) and other lipase-like GDSL-type esterase/lipase motifs. The genomic information provides an understanding of thermophilic genomes and their relevance to stress-tolerant adaptation and explores potential genes, especially for industrial applications.

**Table 6:** Potential genes on the PLS47 genome.

**Feature ID**	**Genes**
Hydratase	E4.3.1.19, ilvA, tdcB; threonine dehydratase [EC:4.3.1.19] nnr; ADP-dependent NAD(P)H-hydrate dehydratase [EC:4.2.1.136 5.1.99.6] kdgD; 5-dehydro-4-deoxyglucarate dehydratase [EC:4.2.1.41] garD; galactarate dehydratase [EC:4.2.1.42] gudD; glucarate dehydratase [EC:4.2.1.40] uxuA; mannonate dehydratase [EC:4.2.1.8] hutU, UROC1; urocanate hydratase [EC:4.2.1.49] acnA; aconitate hydratase A/2-methylisocitrate dehydratase [EC:4.2.1.3 4.2.1.99] E4.3.1.17, sdaA, sdaB, tdcG; L-serine dehydratase [EC:4.3.1.17] K15019; 3-hydroxypropionyl-coenzyme A dehydratase [EC:4.2.1.116]
Dehydrogenase	pdhB; pyruvate dehydrogenase E1 component subunit beta [EC:1.2.4.1] ACADM, acd; acyl-CoA dehydrogenase [EC:1.3.8.7] murB; UDP-N-acetylmuramate dehydrogenase [EC:1.3.1.98] LDH, ldh; L-lactate dehydrogenase [EC:1.1.1.27] lysDH; lysine 6-dehydrogenase [EC:1.4.1.18] HIBADH, mmsB; 3-hydroxyisobutyrate dehydrogenase [EC:1.1.1.31] qorB; NAD(P)H dehydrogenase (quinone) [EC:1.6.5.2] DCAA; acyl-CoA dehydrogenase [EC:1.3.99.-] gudB, rocG; glutamate dehydrogenase [EC:1.4.1.2] ald; alanine dehydrogenase [EC:1.4.1.1]
Peptidase	lepB; signal peptidase I [EC:3.4.21.89] map; methionyl aminopeptidase [EC:3.4.11.18] lytH; peptidoglycan LD-endopeptidase LytH [EC:3.4.-.-] yqgT; g-D-glutamyl-meso-diaminopimelate peptidase [EC:3.4.19.11] pepF, pepB; oligoendopeptidase F [EC:3.4.24.-] pepT; tripeptide aminopeptidase [EC:3.4.11.4] pcp; pyroglutamyl-peptidase [EC:3.4.19.3] dacC, dacA, dacD; serine-type D-Ala-D-Ala carboxypeptidase (penicillin-binding protein 5/6) [EC:3.4.16.4] carboxypeptidase Taq [EC:3.4.17.19] yqgT; g-D-glutamyl-meso-diaminopimelate peptidase [EC:3.4.19.11]
Hydrolase	treC; trehalose-6-phosphate hydrolase [EC:3.2.1.93] ybgC; acyl-CoA thioester hydrolase [EC:3.1.2.-] amhX; amidohydrolase [EC:3.5.1.-] rdgB, ITPA; XTP/dITP diphosphohydrolase [EC:3.6.1.66] gloB, gloC, HAGH; hydroxyacylglutathione hydrolase [EC:3.1.2.6] GCH1, folE; GTP cyclohydrolase IA [EC:3.5.4.16] acyl-CoA hydrolase [EC:3.1.2.20] cbiG; cobalt-precorrin 5A hydrolase [EC:3.7.1.12] Fumarylacetoacetate (FAA) hydrolase family protein Glutathione hydrolase-like YwrD proenzyme
Lipase	GDSL like Lipase 1 GDSL like Lipase 2 Putative Lipase Thermostable monoacylglycerol lipase lip, TGL2; triacylglycerol lipase [EC:3.1.1.3] lipase [Geobacillus kaustophilus] MULTISPECIES: lipase [Geobacillus]
Protease	rseP; regulator of sigma E protease [EC:3.4.24.-] prc, ctpA; carboxyl-terminal processing protease [EC:3.4.21.102] sppA; protease IV [EC:3.4.21.-] clpX, CLPX; ATP-dependent Clp protease ATP-binding subunit ClpX lon; ATP-dependent Lon protease [EC:3.4.21.53] gpr; spore protease [EC:3.4.24.78] gluP; rhomboid protease GluP [EC:3.4.21.105] prsW; protease PrsW [EC:3.4.-.-]
Peroxidase	Putative heme-dependent peroxidase Thiol peroxidase Glutathione peroxidase BsaA katG; catalase-peroxidase [EC:1.11.1.21] catalase/peroxidase HPI [Geobacillus kaustophilus]
Esterase	ymdB; 2',3'-cyclic-nucleotide 2'-phosphodiesterase [EC:3.1.4.16] gdpP; cyclic-di-AMP phosphodiesterase [EC:3.1.4.59] pnbA; para-nitrobenzyl esterase [EC:3.1.1.-] yvaK; carboxylesterase [EC:3.1.1.1] thpR; RNA 2',3'-cyclic 3'-phosphodiesterase [EC:3.1.4.58] pgpH; cyclic-di-AMP phosphodiesterase PgpH [EC:3.1.4.-] glpQ, ugpQ; glycerophosphoryl diester phosphodiesterase [EC:3.1.4.46]

Although this study provides a comprehensive genomic analysis of the thermophilic isolate PLS47, several limitations should be acknowledged. First, the functional roles of candidate genes associated with thermotolerance, stress response, and metabolic pathways were primarily inferred through bioinformatic annotation and comparative analysis. Experimental validation of these genes, including gene expression profiling and enzyme activity assays, was beyond the scope of this study. Additionally, this study focused on a single isolate, which limits broader conclusions regarding genomic diversity and adaptive strategies among thermophiles from the Pria Laot hydrothermal vent. Future studies should incorporate comparative genomic analysis of multiple isolates from the same environment to better elucidate evolutionary adaptations to deep-sea hydrothermal conditions.

To address these limitations, ongoing studies are currently being conducted in our laboratory that focus on the cloning, heterologous expression, purification, and biochemical characterization of predicted hydrolase enzymes, including MAGL lipase and esterase, identified from the PLS47 genome. These studies aim to experimentally validate the extremophilic nature of PLS47 by assessing enzyme thermostability and tolerance to high NaCl concentrations, organic solvents, and alkaline conditions. Further functional characterization of these enzymes, along with plasmid-associated adaptive traits, is expected to provide deeper insights into the molecular basis of extreme tolerance and to support their potential applications in high-temperature and high-salinity industrial bioprocesses.

## 4 Conclusion

The complete genome sequence of PLS47 contains a chromosomal genome of 3,772,236 bp with a G+C content of 52.02%, along with a plasmid of 56,806 bp with a G+C content of 41.06%. A total of 3,708 coding sequences (CDSs) were identified, along with 6 rRNA (5S, 16S, and 23S), 27 rRNA, 88 tRNA genes, and 17 pseudogenes. Comparative genomic analysis based on ANI showed that PLS47 is closely related to *G. thermocatenulatus*. Functional analysis revealed numerous genes encoding enzymes, including proteases, peroxidases, hydrolases, esterases, dehydrogenases, hydratases, and lipases. In addition, the genome exhibits various genes involved in stress-tolerant cell adaptation. This information provides valuable insights into stress-tolerant adaptation and explores potential industrial application genes.

## Data Availability

The datasets presented in this study can be found in online repositories. The names of the repositories and accession numbers can be found in the article.
