# Correction: Genomic analysis of an extremophile PLS47 from a deep-sea vent

**DOI:** 10.3389/fmicb.2026.1903862

**Published:** 2026-06-24

**Authors:** Baiq Repika Nurul Furqan, Made Puspasari Widhiastuty, Febriani, Deviyanthi Nur Afifah, Teuku Mohamad Iqbalsyah, Akhmaloka

**Affiliations:** 1 Biochemistry and Biomolecule Engineering Research Group, Faculty of Mathematics and Natural Sciences, Institut Teknologi Bandung, Bandung, Indonesia; 2 Biomolecules Application Research Group, Chemistry Department, Faculty of Mathematics and Natural Sciences, Syiah Kuala University, Banda Aceh, Indonesia

**Keywords:** deep-sea vent, extremophile, genome, *Geobacillus thermocatenulatus*, PLS47

An incorrect **Funding** statement was provided. The correct **Funding** statement reads:

“The author(s) declared that financial support was received for this work and/or its publication. This work was supported by the research grant from Indonesian Education Scholarship (BPI), Center for Higher Education Funding and Assessment (PPAPT), and Indonesian Endowment Fund for Education (LPDP).”

An incorrect statement was provided. The **Acknowledgments** statement to be changed to: “We would like to thank the Indonesian Education Scholarship (BPI), Center for Higher Education Funding and Assessment (PPAPT), and Indonesian Endowment Fund for Education (LPDP) for providing financial support, which made this research possible.”

The original version of this article has been updated.

